# Brain features of nearly drug‐naïve female monozygotic twins with first‐episode schizophrenia and the classification accuracy of brain feature patterns: A pilot study

**DOI:** 10.1002/brb3.1992

**Published:** 2020-12-08

**Authors:** Tao Zhang, Jie Song, Ce Chen, Ran Li, Yachen Li, Yun Sun, Tao Fang, Weiwei Xu, Hongjun Tian, Chuanjun Zhuo

**Affiliations:** ^1^ Department of Psychiatry Dongying Shengli Hospital Dongying China; ^2^ Department of Psychiatry Shanghai Qingpu District Mental Health Center Shanghai China; ^3^ Department of Psychiatry Wenzhou Seventh Hospital Wenzhou China; ^4^ Psychiatric‐Neuroimaging‐Genetics and Comorbidity Laboratory Tianjin Mental Health Centre Tianjin Anding Hospital Tianjin Medical University Mental Health Teaching Hospital Tianjin China; ^5^ Department of Psychiatry Tianjin Medical University Tianjin China; ^6^ Key Laboratory of Sensory Information Processing Abnormalities in Schizophrenia (SIPP_Lab) Tianjin Fourth Center Hospital Tianjin Fourth Center Hospital Affiliated to Nankai University Tianjin China

**Keywords:** effective connectivity, functional connectivity, gray matter, schizophrenia, twin, white matter

## Abstract

**Background:**

Data on differences in brain features between monozygotic (MZ) twins with and without schizophrenia are scarce.

**Methods:**

We compared brain features of female MZ twins with and without first‐episode schizophrenia and healthy controls (*n* = 20 each). Voxel‐based morphometry and tract‐based spatial statistics were used to analyze differences in brain structure. Whole‐brain effective connectivity (EC) and functional connectivity (FC) networks were constructed using resting‐state functional magnetic resonance imaging (rs‐fMRI) data.

**Results:**

Female twins with schizophrenia exhibited abnormal gray matter volume (GMV) in the basal ganglia and prefrontal and parietal cortices, impairments in the arcuate fasciculus, and significant disruptions (primarily decreases) in nine EC networks. They exhibited rs‐EC alterations involving the limbic areas and subcortex. Combined rs‐EC and rs‐FC data distinguished twins with first‐episode schizophrenia with high accuracy. Combined consideration of structural and functional features enabled the distinction of female MZ twins with schizophrenia from those without schizophrenia and healthy controls with 100% accuracy.

**Conclusions:**

Female MZ twins with schizophrenia exhibited increased GMV, white matter impairment, and disruptions in EC and FC networks. The combination of rs‐EC + rs‐FC data could distinguish female twins with schizophrenia from twins without schizophrenia and healthy controls with 97.4% accuracy, and the addition of structural brain features yielded a 100% accuracy rate. These findings may provide pivotal insight for further study of the mechanisms underlying schizophrenia.

## Graphical abstract

Female monozygotic twins with schizophrenia exhibited decreased GMV in the prefrontal and parietal cortices, increased GMV in the basal ganglia, white matter (i.e., arcuate fasciculus) impairment, and disruptions in EC and FC networks. Female twins with schizophrenia exhibited significant disruptions (primarily decreases) in nine EC networks, most of which involved connections from limbic areas to the subcortex. The combination of rs‐EC and rs‐FC data could be used to distinguish female twins with schizophrenia from twins without schizophrenia and healthy controls with an accuracy of 97.4%, and the addition of structural brain features yielded an accuracy rate of 100%.

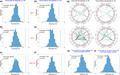

## 1 INTRODUCTION

Over the past 30 years, numerous studies have investigated the brain features of twins, shedding light on brain endophenotypes, genetic and environmental factors influencing schizophrenia, and the reciprocal interaction effect during schizophrenia episodes (Legind, Broberg, Brouwer, et al., 2019; Legind, Broberg, Mandl, et al., 2019). At least 13 notable studies have documented brain structural alterations, and some alterations entailing functional impairment, in twins with schizophrenia (Bohlken et al., 2016; Brans et al., 2008; Bridle et al., 2002; Ettinger et al., 2012; Hedman et al., 2016; Hulshoff Pol et al., 2006; Muller‐Vahl et al., 2007; Picchioni et al., 2017; Rijsdijk et al., 2005; Spaniel et al., 2007; Toulopoulou et al., 2015; van Haren et al., 2004, 2012). For example, Picchioni et al. (2017) noted that the gray matter volume (GMV), white matter volume, and right hippocampal volume were smaller in patients with schizophrenia. Rijsdijk et al. (2005) observed alterations in the hippocampus and third ventricle in twins with schizophrenia. Bohlken et al. (2016) reported that lower global fractional anisotropy was correlated significantly with increased schizophrenia liability. Among monozygotic (MZ) and dizygotic (DZ) twin pairs that were discordant for schizophrenia, Hedman et al. (2016) found progressive global thinning of the cortex, particularly the left superior temporal cortex, in those with schizophrenia. Toulopoulou et al. (2015) revealed that changes in brain volume occur downstream of the schizophrenia liability region and that decreases in brain volume were associated with poor cognitive performance. van Haren et al. (2012) also noted that twins with schizophrenia exhibit alterations in cerebral white matter volume, temporal cortical GMV, and cerebral GMV. Ettinger et al. (2012) observed that schizophrenia was associated with abnormalities in the prefrontal cortex. Brans et al. (2008) revealed that patients with schizophrenia exhibit alterations in the white matter of the frontal, temporal, parietal, and occipital lobes; the cerebellum; and the lateral and third ventricles. In addition, Spaniel et al. (2007) observed reduced cerebral functional activity in the language processing circuit in patients with schizophrenia. Muller‐Vahl et al. (2007) noted that chorea‐acanthocytosis in the striatum, microstructural alterations, and disturbances in metabolism and dopaminergic neurotransmission were related to the manifestation of schizophrenia. Hulshoff Pol et al. (2006) revealed that schizophrenia was associated with decreases in gray matter density, focal increases in white matter density in the left medial orbitofrontal gyrus, and focal decreases in white matter density in the left sensory motor gyrus. van Haren et al. (2004) reported that twin pairs with concordant and discordant schizophrenia exhibited smaller whole‐brain volumes than did control twins. Moreover, the probands of discordant pairs revealed more abnormalities in hippocampal, third ventricular, and lateral ventricular volumes than were observed in concordant twins. Among MZ twin pairs discordant for schizophrenia, Bridle et al. (2002) also noted differences in caudate volumes, but no significant difference in thalamic volume, between affected and unaffected twins.

Simultaneously and more importantly, many notable studies have examined the effects of genetic information on brain alterations in twins with schizophrenia. For example, Narr et al. (2002) proposed that genetic factors, rather than disease‐specific or shared environmental influences, contribute to altered callosal morphology in patients with schizophrenia. Baare et al. (2001) observed that an increased genetic risk of schizophrenia development was related to reduced brain growth in early life. Other decreases in whole‐brain volume or cerebral blood flow have also been observed in patients with schizophrenia (Baare et al., 2001; Goldberg et al., 1994; McNeil et al., 2000).

In recent decades, with the development of advanced magnetic resonance imaging (MRI) techniques, brain features have been characterized in greater detail, further enhancing our understanding of such features in twins with schizophrenia. For example, Weinberger et al. and Bartley et al. (Bartley et al., 1993; Casanova, Zito, Goldberg, Suddath, et al., 1990) revealed that greater differences in left hippocampal volume between affected and unaffected twins were associated with greater differences in prefrontal physiological activation during the Wisconsin Card Sorting Test. In the affected twin group, prefrontal activation was related strongly to the left and right hippocampal volumes, typifying the asymmetric model (Bartley et al., 1993; Casanova, Zito, Goldberg, Suddath, et al., 1990). Corpus callosum curvature has also been observed in twins with schizophrenia, and the shapes of the anterior and middle corpus callosum segments have been found to be influenced by sex in these twins (Casanova, Sanders, Goldberg, et al., 1990; Casanova, Zito, Goldberg, Suddath, et al., 1990). Suddath et al. (1990) reported that twins with schizophrenia exhibited larger lateral and third ventricle volumes than did their healthy counterparts. Casanova, Zito, Goldberg, Abi‐Dargham, et al. (1990) further reported corpus callosum abnormalities in MZ twins discordant for schizophrenia.

Despite the outstanding quality of these previous findings, some discrepancies warrant further exploration. They may be related to differences in the samples investigated (e.g., first episode vs. chronic schizophrenia, MZ vs. DZ twins) or MRI techniques used (e.g., 1.5 or 3.0 T, processing methods). Advances in MRI techniques over the last 10 years have improved the precision of structural and functional brain analyses (Glasser et al., 2016; Setsompop et al., 2013). However, few researchers have utilized these methods to study twins with and without schizophrenia. Furthermore, differences and similarities between MZ twins with and without schizophrenia, and between DZ twins with and without schizophrenia, remain to be fully elucidated.

To address these issues, we conducted a pilot study to investigate brain structural and functional alterations in female MZ twin pairs with discordant schizophrenia and in healthy controls. We applied advanced analyses of resting‐state functional magnetic resonance imaging (rs‐fMRI) data to classify brain structure and function in these three twin groups. We hypothesized that female MZ twins with and without schizophrenia would exhibit structural and functional brain alterations that can be used as markers for the early identification of schizophrenia.

## 2 METHODS

Effective connectivity (EC) refers to the causal (thus directional) influence of neural activity in one region on that in another region (Chao‐Gan & Yu‐Feng, 2010; Gong, Puthusseryppady, et al., 2019; Kottaram et al., 2018; Mastrovito et al., 2018; Rozycki et al., 2018; Wang et al., 2015). Functional connectivity (FC) refers to the instantaneous (i.e., zero‐time lagged) temporal correlation (thus unidirectional) between spatially distinct brain regions(Palaniyappan et al., 2013). We performed an advanced resting‐state effective connectivity (rs‐EC) (Gong, Puthusseryppady, et al., 2019; Mastrovito et al., 2018) analysis to construct whole‐brain EC networks via Granger causality analysis (GCA) of rs‐fMRI data. We compared brain‐wise EC among 20 female MZ twin pairs with and without first‐episode schizophrenia and 20 healthy female MZ twins. Because EC and FC measure inter‐regional interactions in different ways, we performed a similar rs‐FC (Kottaram et al., 2018) analysis to identify differences in the ways in which FC and EC capture abnormalities of brain connectivity caused by schizophrenia. To test the discriminative power of the whole‐brain EC pattern as a neuroimaging marker for the diagnosis of schizophrenia, we performed multivariate pattern analysis (MVPA) (Fan et al., 2016; Gong, Cheng, et al., 2019; Rozycki et al., 2018; Yuan et al., 2016) to classify patients and controls based on whole‐brain EC, FC, and their combination. Based on this classification, we adopted voxel‐based morphometry (Torres et al., 2016) and tract‐based spatial statistics (Bopp et al., 2017) to investigate differences in brain structure among the three twin groups.

### 2.1 Participants

The database utilized in the present study was established at Tianjin Mental Health Centre, Tianjin, China, in 2011 (Huang et al., 2019; Xu et al., 2017; Yin et al., 2017, 2018). It contains data on approximately 40,000 patients with schizophrenia, recorded via our hospital's voluntary management system. Following Xu, Li, et al. (2017), the inclusion criteria for this study were as follows: female sex (due to MRI compliance); MZ twin status; age 28–35 years; right‐handedness; ability to comply with the MRI examination, with no MRI contraindication; no history of substance abuse; for healthy controls, no psychiatric disorder or first‐degree relative with a psychotic disorder; and, for twin pairs, first‐episode schizophrenia with continuous standard treatment in the previous 6 months (≤2 months prior to enrollment) and discontinuation of antipsychotic treatment ≥2 weeks prior to MRI examination in one twin, and no schizophrenia or other psychiatric disorder in the other twin. Healthy control twins were recruited from 26 communities using award‐winning advertisements.

Experienced psychiatrists examined all patients and made diagnoses using the Structured Clinical Interview from the fourth edition of the Diagnostic and Statistical Manual of Mental Disorders; no participating patient had any other systemic disease, chronic condition, or head trauma. The severity of schizophrenia symptoms was quantified using the Positive and Negative Syndrome Scale (PANSS) (Aboraya & Nasrallah, 2016).

The Ethics Committees of Tianjin Mental Health Centre approved this study (approval no. TJMH‐Cohort Study‐004). Written informed consent was obtained from all participants and their legal guardians (as per the Chinese requirement) prior to data acquisition.

### 2.2 MRI data acquisition, preprocessing, and processing

Whole‐brain sagittal three‐dimensional T1‐weighted structural images (voxel size, 1 × 1 × 1 mm^3^) and resting‐state functional images (repetition time, 2,000 ms; in‐plane resolution, 3.4375 × 3.4375 mm^2^; slice thickness, 4 mm; duration, 6 min) were acquired using a 3.0‐T MRI scanner (Discovery MR750; General Electric). Sponges and earplugs were used to reduce head motion and noises, respectively. Sagittal 3D T1‐weighted structural images were acquired using the following imaging parameters: repetition time (TR) = 8.2 ms; echo time (TE) = 3.2 ms; inversion time (TI) = 450 ms; flip angle (FA) = 12°; field of view (FOV) = 256 × 256 mm; matrix = 256 × 256; slice thickness = 1 mm; gap = 0 mm; and number of slices = 188. Resting‐state functional images were obtained using a gradient‐echo single‐shot Echo Planar Imaging sequence with the following parameters: TR/TE = 2,000/45 ms; FOV = 220 × 220 mm; matrix = 64 × 64; FA = 90°; slice thickness = 4 mm; gap = 0.5 mm; 32 interleaved transverse slices; and duration = 6 min (resulting in 180 volumes). All participants were instructed to keep their eyes closed and stay awake during the fMRI scanning.

The rs‐fMRI data were preprocessed using the Data Processing Assistant for Resting‐State fMRI (DPARSF) software package (http://rfmri.org/DPARSF) (Chao‐Gan & Yu‐Feng, 2010). The preprocessing steps included discarding of the first 10 volumes, slice timing, realignment, spatial normalization, and smoothing (see the Supporting information for further details). The signals of each voxel were also cleaned by out‐regressing of nuisance covariates (including 24 head motion parameters (Yuan et al., 2016), white matter, cerebrospinal fluid, and global signals), band‐pass filtering (0.01–0.1 Hz), and linear detrending. Subsequently, the resting‐state fMRI data were preprocessed using the software package DPARSF (Data Processing Assistant for Resting‐State fMRI; http://rfmri.org/DPARSF) with the following steps. To reach the equilibrium of signal and allow patients to adapt to the scanning environment, the first 10 volumes were discarded. Then, signals of each slice were corrected for the difference in acquisition time between slices within each volume. Realignment between volumes was performed to correct for head motion. Participants with excessive head motion (>2 mm displacement in any axis or > 2° rotation around any axis) were discarded in subsequent analyses. The realigned images were coregistered to the structural T1 image, then normalized to the standard Montreal Neurological Institute space using the unified segmentation procedure, and also resampled to 3 × 3 × 3 mm^3^ voxel size. The normalized and resampled images were spatially smoothed using a Gaussian kernel of 8 mm full‐width at half‐maximum.

### 2.3 Whole‐brain parcellation

Parcellation of the cerebrum was performed according to the Brainnetome Atlas (http://atlas.brainnetome.org/bnatlas.html) (Fan et al., 2016), which divides the gray matter of the cerebrum into 116 subregions. The Anatomical Automatic Labeling Atlas (Tzourio‐Mazoyer et al., 2002) was used to define the subregions of the cerebellum. The fMRI time series of all voxels in each region was averaged to obtain an average regional time series.

### 2.4 Construction of EC and FC networks

EC and FC analyses were performed using the REST software package (www.restfmri.net). The bivariate coefficient‐based GCA approach (Chen et al., 2011) (based on an autoregressive model with a time lag order of 1) was adopted to investigate the causal relationship between each pair of brain regions, resulting in the creation of a whole‐brain EC network for each participant. These causal relationships were estimated by assessing whether previous signals of the fMRI time series in region A could predict the current signal of the time series in region B (i.e., whether the activity in A caused the activity in B). Similarly, a whole‐brain FC network was created for each participant by estimating FC between each pair of regions using Pearson's coefficients of correlation between the average time series of these two regions.

### 2.5 Permutation procedures in the univariate analyses

The statistical significance of the presence of each rs‐EC (from region A to region B, or vice versa) in each group (patient group and control group) was determined by permutation test (*n* = 1,000) and corrected for multiple comparisons (*p* < .05, corrected). More specifically, in each group, a *t* value was obtained for each rs‐EC using a one‐sample *t* test and the corresponding *p* value of each rs‐EC was determined using the following procedure: (a) For each group, the values of a given rs‐EC of all participants were randomly assigned with a positive or negative sign and then were entered into a one‐sample *t* test to obtain a *t* value; (b) the first step was performed for each of the 73,712 rs‐ECs, resulting in 73,712 *t* values; (c) all *t* values were converted into absolute values and the maximal absolute *t* value was taken for subsequent steps; (d) the above steps were repeated 1,000 times, resulting in 1,000 maximal absolute *t* values which were used to build a null distribution of the absolute *t* values obtained at chance level; and (e) the true *t* value of each rs‐EC (i.e., the *t* value obtained without randomly changing the sign of the rs‐EC) was converted into the absolute value and then compared with the null distribution of the chance‐level absolute *t* values to generate a corrected *p* value of each rs‐EC—the corrected *p* value was calculated as the percentage of the chance‐level absolute *t* values that were equal to or greater than the true absolute *t* value. The sign of each rs‐EC at group level was further determined by the sign of the true *t* value of the given rs‐EC.

### 2.6 Univariate analysis

Whether a given rs‐EC was significantly different between patient group and control group was determined by a permutation test (*p* < .05, corrected) similar to the above permutation test procedure with only two exceptions: (a) The true *t* value or chance‐level *t* values were obtained from two‐sample *t* tests rather than one‐sample *t* tests; and (b) to generate the chance‐level *t* values, the labels of participants (i.e., patient or control) were randomly permutated across all participants (i.e., randomly assign each participant to patient group or control group with the restriction of keeping the number of patients or controls unchanged) rather than randomly changing the sign of each rs‐EC.

Whether a given rs‐EC was significantly different between patient group and control group was determined by a permutation test (*p* < .05, corrected) similar to the above permutation test procedure with only two exceptions: (a) The true *t* value or chance‐level *t* values were obtained from two‐sample *t* tests rather than one‐sample *t* tests; and (b) to generate the chance‐level *t* values, the labels of participants (i.e., patient or control) were randomly permutated across all participants (i.e., randomly assign each participant to patient group or control group with the restriction of keeping the number of patients or controls unchanged) rather than randomly changing the sign of each rs‐EC.

### 2.7 MVPA

We performed MVPA using an in‐house MATLAB script to investigate whether the spatial patterns of whole‐brain EC (considering all rs‐EC variables simultaneously) could discriminate patients with schizophrenia from controls. MVPA is a machine‐learning technique by which patterns are examined to solve a classification problem. We used a support vector machine to identify a classification hyperplane between patients and controls. All participant data were split into training and test datasets using a leave‐one‐out cross‐validation (LOOCV) procedure, and the final classification accuracy was determined based on the percentage of correctly classified participants across all LOOCV steps. The statistical significance of the classification accuracy was determined by a permutation test (*n* = 1,000) as follows: (a) The class labels of all participants were randomly permutated for 1,000 times to generate 1,000 chance‐level classification accuracies (each permutation generates one chance‐level accuracy); and (b) the 1,000 chance‐level classification accuracies were used to build a null distribution of the chance‐level accuracies with which the true classification accuracy (i.e., the accuracy obtained based on the true class labels) was compared to generate the *p* value (i.e., the percentage of the chance‐level accuracies that are equal to or greater than the true accuracy). The significance threshold was set to *p* < .05. We also extracted the mean weight of each rs‐EC variable across all LOOCV steps to quantify its contribution to the overall classification accuracy. To characterize EC networks with large contributions, we further extracted the top 1% of ECs with the highest absolute weights and examined whether they overlapped with those exhibiting significant alterations in the univariate analysis.

The same MVPA procedure was performed using whole‐brain FC data, as well as the combination of EC and FC data, to investigate whether the spatial patterns of whole‐brain FC could discriminate patients from controls and whether the combination of the two could improve classification performance, respectively.

### 2.8 Ethical approval

The Ethics Committees of Tianjin Mental Health Centre approved this study (approval no. TJMH‐Cohort Study‐004). All procedures performed in studies involving human participants were in accordance with the ethics standards of the institutional and national research committee and with the 1964 Helsinki Declaration and its later amendments or comparable ethics standards.

## 3 RESULTS

### 3.1 Demographic and clinical characteristics of participants

After image quality control, data from 20 female MZ twin pairs who were discordant for schizophrenia and 20 healthy control female MZ twins were included in the final analyses. The demographic and clinical characteristics of the participants are shown in Table 1. Age did not differ among the three groups, but the groups exhibited significant differences in PANSS, MATRICS Consensus Cognitive Battery (MCCB, including speed processing, attention, working memory, verbal learning, visual learning, problem reasoning, social cognition), and Global Assessment Of Functioning (GAF) scores and levels of education.

**Table 1:** Demographic and clinical characteristics of participating female monozygotic twins

Variable	Healthy control female monozygotic twins *N* = 20	Female monozygotic twins without schizophrenia *N* = 20	Female monozygotic twins with schizophrenia *N* = 20	*F/t*	*p*
Age, years, mean (*SD*)	23.0 (2.4)	24.2 (3.0)	23.9 (3.5)	0.866	.430
Education level, years, mean (*SD*)	16.1 (2.5)	15.0 (3.0)	10.0 (3.0)	26.15	<.001
Duration of illness, Months, mean (*SD*)	N/A	N/A	5.3 (1.9)	N/A	N/A
PANSS score, mean (*SD*)	N/A	34.2 (1.5)	67.1 (6.8)	−21.13	<.001
MCCB score, mean (*SD*)					
Speed processing	46.9 (8.4)	40.4 (11.2)	34.5 (7.5)	9.15	<.001
Attention	46.3 (10.2)	44.7 (7.3)	30.3 (10.9)	16.87	<.001
Working memory	48.8 (8.2)	42. 5 (9.6)	34.1 (12.4)	10.42	<.001
Verbal learning	48.1 (10.1)	36.4 (11.6)	34.4 (10.4)	9.53	<.001
Visual learning	45.0 (10.1)	51.2 (8.7)	42.1 (8.8)	5.08	.009
Problem reasoning	44.4 (7.6)	46.5 (10.5)	37.0 (10.4)	5.41	.007
Social cognition	44.5 (9.6)	40.8 (14.0)	35.7 (12.4)	2.65	.079
GAF	98.5 (8.6)	86.0 (7.5)	78.0 (7.6)	34.07	<.001

Note

Data are presented as mean (*SD*).

Abbreviations: GAF, Global Assessment of Functioning; MCCB, MATRICS Consensus Cognitive Battery; PANSS, Positive and Negative Syndrome Scale.

### 3.2 Differences in brain structure

**Figure 1:**
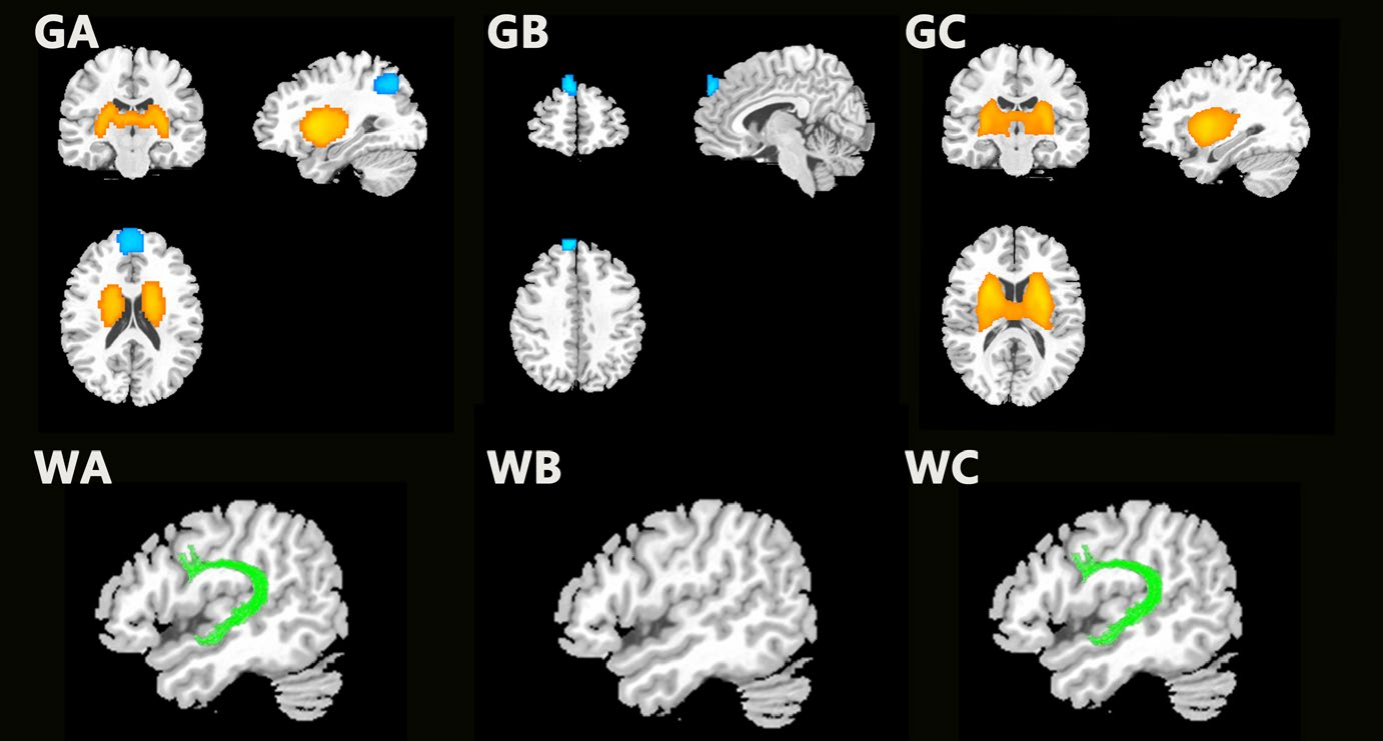
Gray matter (G) and white matter (W) alterations in female monozygotic twins with and without schizophrenia, compared with healthy controls

Compared with healthy controls, twins with schizophrenia exhibited increased GMV in the basal ganglia and decreased GMV in the prefrontal and parietal lobes (Figure 1: GA); twins without schizophrenia exhibited decreased GMV in components of the prefrontal lobe (Figure 1: GB). Basal ganglia volumes were greater in twins with than in those without schizophrenia (Figure 1: GC). Compared with healthy controls, twins with schizophrenia exhibited abnormalities in the arcuate fasciculus (Figure 1: WA); twins without schizophrenia exhibited no significant white matter alteration (Figure 1: WB). Notably, the abnormalities in the arcuate fasciculus also represented a significant difference between twins with and without schizophrenia (Figure 1: WC; FWE‐corrected *p* < .001).

### 3.3 Differences in brain function

**Figure 2:**
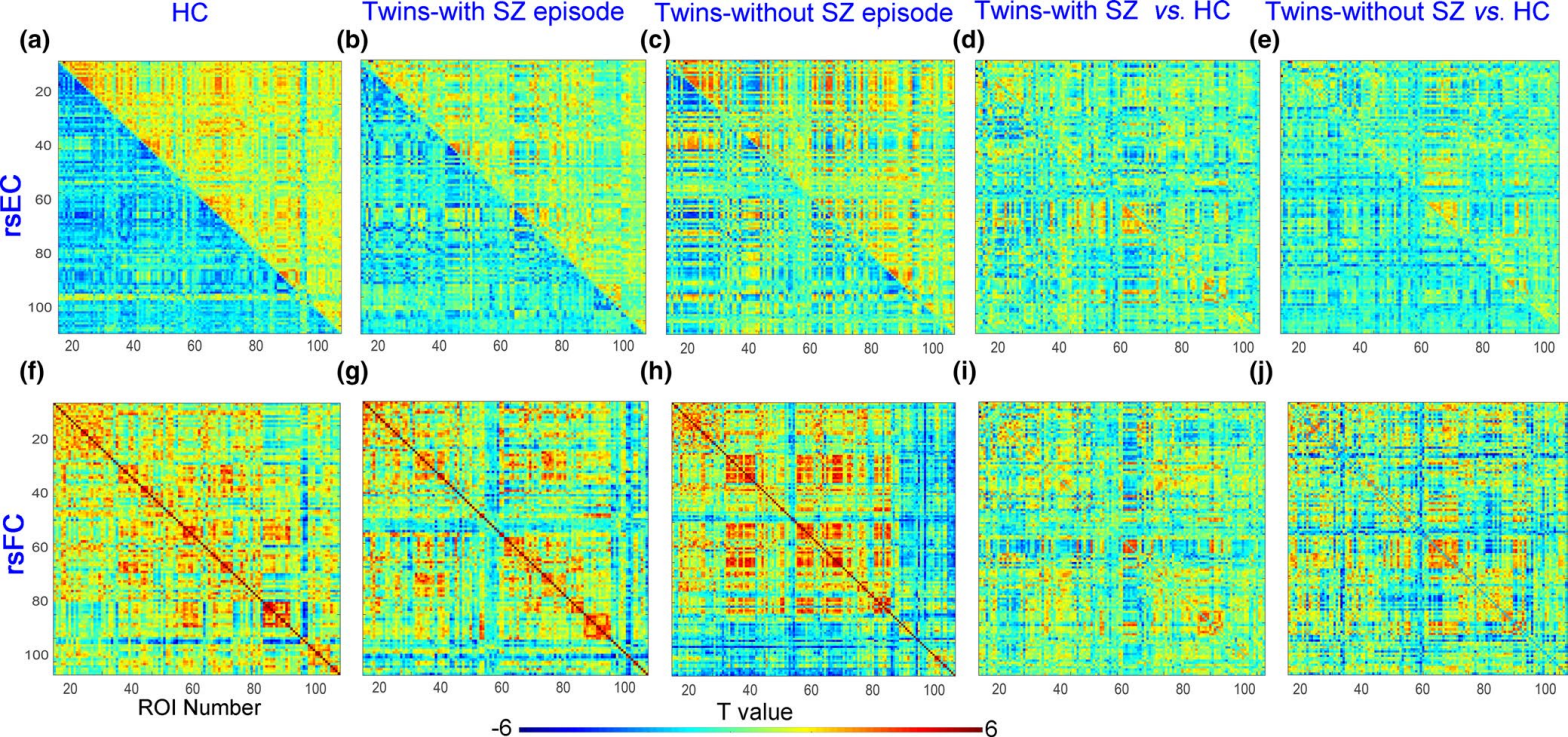
Resting‐state effective connectivity (rs‐EC) and resting‐state functional connectivity (rs‐FC) matrices for healthy controls (HC; a, f), twins with schizophrenia (SZ; b, g), and twins without SZ (c, h), and differences in rs‐EC (d, i) and rs‐FC (e, j) from HCs. Each matrix entry indicates a connection. Colors indicate *t* values. ROI, region of interest

We identified 157 significant EC variables and 359 significant FC variables in the entire study group (Figure 2a–j). The differences in EC and FC between patients and controls are shown in Figure 2d,i, respectively. Significant differences in EC and FC in twins with schizophrenia did not correlate with the total PANSS score, MCCB subitem scores, or the total GAF score.

**Figure 3:**
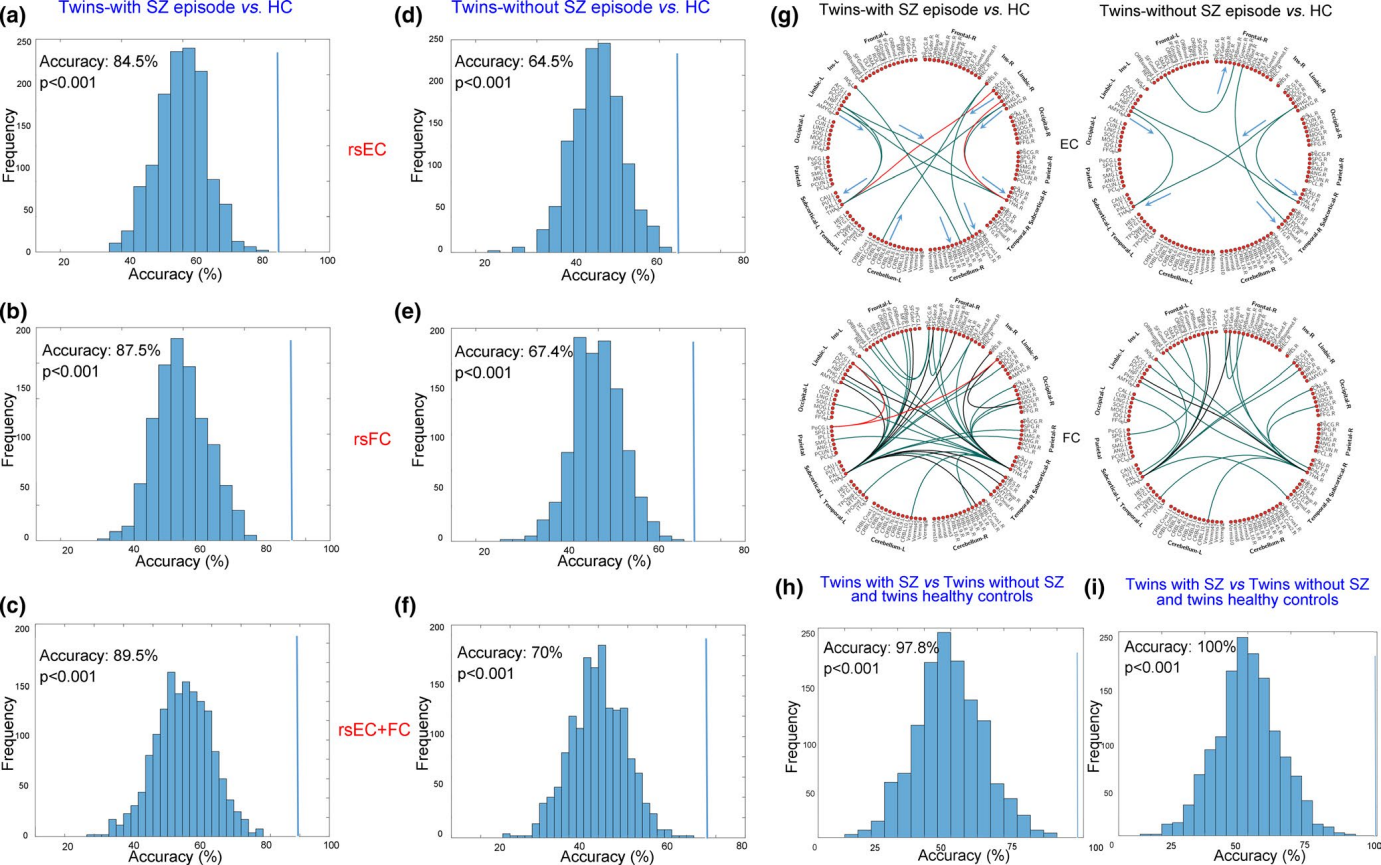
Multivariate pattern analysis results for the classification of twins with and without schizophrenia (SZ) vs. healthy controls (HC) using whole‐brain resting‐state effective connectivity (rs‐EC; a, d), resting‐state functional connectivity (rs‐FC; b, e), and their combination (c, f). Spatial distributions of rs‐EC (g, top) and rs‐FC (g, bottom); colors indicate significant differences in rs‐EC (blue, decreased in patients; red, increased in patients). Results for twins with SZ vs. twins without SZ and HCs (h, rs‐EC + rs‐FC; i, rs‐EC + rs‐FC + structural alterations). *p*‐values were calculated as the proportion of permutations (of 1,000) with accuracy ≥actual classification accuracy (for 0, *p* < .001)

Nine EC variables differed significantly between patients and controls, and six EC variables differed significantly between twins without schizophrenia and controls (Figure 3g). Notably, the nine EC variables in twins with schizophrenia involved mainly connections from limbic areas (including the hippocampus, parahippocampus, and cingulate gyrus) to the subcortex (including the putamen, caudate, and thalamus), from the insula to the cerebellum, from the left frontal cortex to the right frontal cortex, and from the right frontal lobe to the right temporal cortex. Two of these variables had positive values in both groups, but significantly lower values in patients, and seven variables had positive values in the control group and negative values in the patient group.

The numbers of significant rs‐FC variables differed among the three groups. Notably, none of them involved a connection identified as abnormal based on rs‐EC, indicating that rs‐FC and rs‐EC capture different information regarding the relationships between pairs of brain regions. Abnormal rs‐FC was distributed widely across the whole brain in twins with and without schizophrenia (Figure 3g, bottom).

### 3.4 MVPA results

The classification accuracies for whole‐brain rs‐EC data (84.5%), whole‐brain rs‐FC data (87.5%), and their combination (89.5%), and the corresponding null distributions, revealed that twins with schizophrenia were successfully distinguished from healthy controls (Figure 3). Twins without schizophrenia were successfully distinguished from healthy controls with accuracies of 64.5% (rs‐EC), 64.7% (rs‐FC), and 70% (rs‐EC + rs‐FC; Figure 3). Using the combined data, twins with and without schizophrenia were successfully distinguished with an accuracy of 97.8% (Figure 3). When structural alterations were added to the combined data, twins with schizophrenia were successfully distinguished from twins without schizophrenia and healthy controls with an accuracy of 100%. All accuracy values were significantly higher than those attributable to chance (*p* < .001).

## 4 DISCUSSION

To our knowledge, the present study is the first to explore structural and functional brain alterations in nearly drug‐naïve female MZ twins with and without first‐episode schizophrenia, as well as the accuracy of classifying patients and controls based on these alterations. Twins with schizophrenia exhibited distinct increases and reductions in specific brain volumes, arcuate fasciculus impairment, and distinct EC patterns. Our findings further indicated that the combination of rs‐EC and rs‐FC data could be used to distinguish twins with first‐episode schizophrenia from those without schizophrenia and healthy controls with high accuracy, and that the additional consideration of structural alterations increased the accuracy to 100%.

Consistent with the traditional basal ganglia–dopamine hypothesis, we observed increases in the size of the basal ganglia in the patient group, which may underlie the positive symptoms of schizophrenia (Howes & Shitij, 2009; Kambeitz et al., 2018; Martino et al., 2018; McCutcheon et al., 2019). Previous studies have also detected changes in the basal ganglia and prefrontal/parietal cortices in patients with schizophrenia (Camchong et al., 2006; Fusar‐Poli et al., 2010; McCutcheon et al., 2019; Scheuerecker et al., 2009; Shenton et al., 2001). In accordance with our findings, studies supporting the neurodevelopmental hypothesis of schizophrenia have also documented decreases in prefrontal and parietal cortex volume, which may be related to the negative symptoms of schizophrenia (Cancel et al., 2019; Gong et al., 2016; Murray et al., 2017; Smieskova et al., 2013; Yan et al., 2019). Arcuate fasciculus impairment in patients with schizophrenia has also been reported previously (Behdinan et al., 2015; Dietsche et al., 2017; Jiang et al., 2019; Kanaan et al., 2005; Kelly et al., 2018; Peters et al., 2008; Voineskos et al., 2013).

In the present study, almost all rs‐EC abnormalities in twins with schizophrenia were spatially restricted to pathways from limbic areas to the basal ganglia. This altered rs‐EC involved a wide range of basal ganglia subregions, consistent with the results of recent studies focusing on subregions of the basal ganglia in schizophrenia (Allen et al., 2016; Beckmann & Lauer, 1997; Bostan et al., 2013; Cohen & Frank, 2009; Ebdrup et al., 2010; Emma et al., 2010; Mccarley et al., 1999; Mccutcheon et al., 2017; Rich et al., 2016; Sim et al., 2006; Torres et al., 2016). Taken together, these findings suggest that disruptions in the flow of information from these limbic areas to the subcortex play key roles in the pathophysiology of schizophrenia. The limbic areas and basal ganglia are known to act as relay stations, playing important roles in the bidirectional transmission of neuronal signals into or out of cortical areas, including those of the limbic system (Shipp, 2017). Furthermore, all areas identified are known to exhibit structural and/or functional alterations in patients with schizophrenia. In accordance with previous hypotheses, our findings support the notion that rs‐EC from the insula to the cerebellum, from the left to the right frontal cortex, and from the right frontal lobe to the right temporal cortex plays a key role in information communication in schizophrenia (Berman et al., 2013; Dong et al., 2017; Peters et al., 2016; Tol et al., 2014). Ultimately, the accumulated evidence suggests that the pathophysiology of schizophrenia involves disruptions in rs‐EC, which may further explain symptoms of schizophrenia such as delusions, hallucinations, disordered thoughts, and mood disorders (Berman et al., 2013; Dong et al., 2017; Mizuno et al., 2016; Tol et al., 2014; Valton et al., 2017). Given that all rs‐ECs identified also play roles in cognition, emotion, and executive function (Allen et al., 2016; Beckmann & Lauer, 1997; Berman et al., 2013; Bostan et al., 2013; Cohen & Frank, 2009; Dong et al., 2017; Ebdrup et al., 2010; Emma et al., 2010; Kelly et al., 2018; Mccarley et al., 1999; Mccutcheon et al., 2017; Mizuno et al., 2016; Peters et al., 2016; Rich et al., 2016; Shipp, 2017; Sim et al., 2006; Tol et al., 2014; Torres et al., 2016; Valton et al., 2017), our findings further suggest that schizophrenia involves impairment of these networks.

### 4.1 Distinguishing female MZ twins with first‐episode schizophrenia from twins without schizophrenia and healthy controls using whole‐brain rs‐EC and rs‐FC patterns

Clinical diagnoses of schizophrenia rely mainly on subjective assessments based on questionnaire responses and psychiatrists’ experience, and are thus of questionable reliability. Therefore, researchers have aimed to develop neuroimaging tools for the objective diagnosis of schizophrenia. The ability to successfully distinguish patients and controls based on whole‐brain patterns in the present study suggests that patients with schizophrenia exhibit characteristic rs‐EC and rs‐FC relative to healthy controls. Importantly, the combined use of rs‐FC and rs‐EC data further improved the classification accuracy, confirming that these data capture different and complementary characteristics of the functional dysconnectivity associated with schizophrenia. Our results suggest that the consideration of rs‐EC will aid the development of diagnostic neuroimaging tools for schizophrenia.

Moreover, although two of the nine rs‐EC variables identified in the univariate analysis provided the greatest contributions (within the top 1%) to the classification of patients and controls, the overall pattern utilized by the classifier differed markedly from the univariate pattern of rs‐EC alterations. The observed rs‐EC weight map indicates that rs‐EC alterations associated with schizophrenia are likely distributed more diffusely in the brain than detected by traditional univariate statistical tests. Such tests control only for false‐positive error, and thus may not account for high false‐negative errors (i.e., many rs‐EC alterations may remain undetected), whereas MVPA takes advantage of any information useful for improving classification performance (i.e., even weak differences in rs‐EC between groups). However, some significant rs‐EC alterations may have had low weights because they contained redundant information and were thus not used by the classifier. Taken together, our results suggest that multivariate and univariate approaches provide different information and that the two methods should be used in conjunction to more fully elucidate rs‐EC alterations caused by schizophrenia.

Although differences in brain structural and functional features between affected and unaffected first‐degree relatives of patients with schizophrenia have been reported, most reports describe minor functional alterations with only slight regional overlap between affected and unaffected relatives, and no structural alterations in the gray matter in unaffected relatives (Saarinen et al., 2020). The differences in rs‐EC and rs‐FC that we observed between affected and unaffected twins provide new evidence for brain functional (but not structural) alteration in the first‐degree relatives of patients with schizophrenia. These findings remind us to pay more attention to early brain functional alterations in unaffected twins to protect against the development of schizophrenia.

Many studies have shown that gene–environment interaction is a strong risk factor for the development of schizophrenia in siblings of patients with this disease (van Os et al., 2020). Heritability and environmental factors each account for about half of the phenotypic variance of schizophrenia (Chou et al., 2017), and thus should be considered simultaneously in the development of tailored prevention strategies for the siblings of patients with schizophrenia. As environmental factors can be improved more easily than heritability factors, attention to the living environment in individuals’ early years would be especially useful.

### 4.2 Limitations

This pilot study has several limitations. First, as our results were derived from nearly drug‐naïve patients (who discontinued treatment 2 weeks prior to MRI examination), we cannot exclude the potential confounding influence of antipsychotic treatment in the previous 6 months. In addition, to ensure compliance during MRI examination, we included only female patients and MZ twins. Thus, further studies are required to determine whether similar rs‐EC patterns exist in male patients and DZ twins. Second, we assumed that the lives of the female MZ twins were similar, and did not assess the effects of personality or stressors on the early differential diagnosis of this disease. Such confounding factors may have important effects on patients, and should be considered in future research. Third, the sample examined in this pilot study was small, which may have led to overestimation of our algorithm's accuracy; this accuracy should be validated in large multicenter studies.

## 5 CONCLUSIONS

In the present study, female MZ twins with schizophrenia exhibited decreased GMV in the prefrontal and parietal cortices, increased GMV in the basal ganglia, white matter (i.e., arcuate fasciculus) impairment, and disruptions in EC and FC networks. In addition, female twins with schizophrenia exhibited significant disruptions (primarily decreases) in nine EC networks, most of which involved connections from limbic areas to the subcortex. The combination of rs‐EC and rs‐FC data could be used to distinguish female twins with schizophrenia from twins without schizophrenia and healthy controls with an accuracy of 97.4%, and the addition of structural brain features yielded an accuracy rate of 100%. Our findings may aid elucidation of the mechanisms underlying schizophrenia and the development of diagnostic neuroimaging markers to distinguish among twins with and without schizophrenia and healthy controls.

## CONFLICT OF INTEREST

None declared..

## AUTHOR CONTRIBUTIONS

All authors listed have made a substantial, direct, and intellectual contribution to the work, and approved it for publication.

## INFORMED CONSENT

Written informed consent was obtained from all participants and their legal guardians (as per the Chinese requirement) prior to data acquisition.s

### Peer Review

The peer review history for this article is available at https://publons.com/publon/10.1002/brb3.1992.

## Supporting information

Supporting Information

## Data Availability

Data are available from the corresponding author upon reasonable request.
