# Supplementary material for: Brain features of nearly drug‐naïve female monozygotic twins with first‐episode schizophrenia and the classification accuracy of brain feature patterns: A pilot study
Source: Brain Behav. 2020 Dec 8;11(2):e01992. doi: 10.1002/brb3.1992 (PMC7882158; doi:10.1002/brb3.1992)
Supplement: Supplementary file 1 — Supporting Information [file BRB3-11-e01992-s001.doc]

**Supplementary materials**

**MRI data acquisition**

The whole brain images were acquired using a 3.0T MR scanner (Discovery MR750, General Electric, Milwaukee, WI, USA). Sponges and earplugs were used to reduce head motion and noises, respectively. Sagittal 3D T1-weighted structural images were acquired using the following imaging parameters: repetition time (TR) = 8.2 ms; echo time (TE) = 3.2 ms; inversion time (TI) = 450 ms; flip angle (FA) = 12°; field of view (FOV) = 256 × 256 mm; matrix = 256 × 256; slice thickness = 1 mm, gap = 0 mm, and number of slices = 188. Resting-state functional images were obtained using a gradient-echo single-shot EPI (Echo Planar Imaging) sequence with the following parameters: TR/TE = 2000/45 ms; FOV = 220 × 220 mm; matrix = 64 × 64; FA = 90°; slice thickness = 4 mm; gap = 0.5 mm; 32 interleaved transverse slices, duration = 6 mins (resulting in 180 volumes). All participants were instructed to keep their eyes closed and stay awake during the fMRI scanning.

**Data preprocessing**

The resting-state fMRI data were preprocessed using the software package DPARSF (Data Processing Assistant for Resting-State fMRI; <http://rfmri.org/DPARSF>) with the following steps. To reach the equilibrium of signal and allow patients to adapt to the scanning environment, the first 10 volumes were discarded. Then, signals of each slice were corrected for the difference in acquisition time between slices within each volume. Realignment between volumes was performed to correct for head motion. Participants with excessive head motion (>2mm displacement in any axis or >2° rotation around any axis) were discarded in subsequent analyses. The realigned images were coregistered to the structural T1 image, then normalized to the standard MNI (Montreal Neurological Institute) space using the unified segmentation procedure and also resampled to 3×3×3 mm^3^ voxel size. The normalized and resampled images were spatially smoothed using a Gaussian kernel of 8 mm full-width at half-maximum (FWHM).

**Permutation procedures in the** **univariate analyses**

The statistical significance of the presence of each rs-EC (from region A to region B, or vice versa) in each group (patient group and control group) was determined by permutation test (n=1000) and corrected for multiple comparisons (P<0.05, corrected). More specifically, in each group, a t value was obtained for each rs-EC using a one-sample t test and the corresponding p value of each rs-EC was determined using the following procedure: (1) for each group, the values of a given rs-EC of all participants were randomly assigned with a positive or negative sign and then were entered into a one-sample t test to obtain a t value; (2) the first step was performed for each of the 73712 rs-ECs, resulting in 73712 t values; (3) all t values were converted into absolute values and the maximal absolute t value was taken for subsequent steps; (4) the above steps were repeated 1,000 times, resulting in 1,000 maximal absolute t values which were used to build a null distribution of the absolute t values obtained at chance level; (5) the true t value of each rs-EC (i.e., the t value obtained without randomly changing the sign of the rs-EC) was converted into the absolute value and then compared with the null distribution of the chance-level absolute t values to generate a corrected P value of each rs-EC – the corrected P value was calculated as the percentage of the chance-level absolute t values that were equal to or greater than the true absolute t value. The sign of each rs-EC at group level was further determined by the sign of the true t value of the given rs-EC.

Whether a given rs-EC was significantly different between patient group and control group was determined by a permutation test (P<0.05, corrected) similar to the above permutation test procedure with only two exceptions: (1) the true t value or chance-level t values were obtained from two-sample t tests rather than one-sample t-tests; (2) to generate the chance-level t values, the labels of participants (i.e., patient or control) were randomly permutated across all participants (i.e., randomly assign each participant to patient group or control group with the restriction of keeping the number of patients or controls unchanged) rather than randomly changing the sign of each rs-EC.

**Permutation procedures in the MVPA**

The statistical significance of the classification accuracy was determined by a permutation test (n=1000) as follows: (1) the class labels of all participants were randomly permutated for 1000 times to generate 1000 chance-level classification accuracies (each permutation generates one chance-level accuracy); (2) the 1000 chance-level classification accuracies were used to build a null distribution of the chance-level accuracies with which the true classification accuracy (i.e., the accuracy obtained based on the true class labels) was compared to generate the P value (i.e., the percentage of the chance-level accuracies that are equal to or greater than the true accuracy). The significance threshold was set to P<0.05.
